# Christmas Cheer

**Published:** 1893-12-23

**Authors:** 


### CHRISTMAS CHEER.

' Cheer" is eminently an English idea. "Cheer, Boys, Cheer," was a fine title for an English song. "Let us give him a good ringing cheer" is the natural expression of an Englishman when he "lets his heart go" for his favourite soldier or politician. We are not a volatile or a frisky people by any means; but we are certainly cheery—cheery, hopeful, and determined.

Business men tell us we have fallen upon bad times just now—very bad times; and that appears to be true. Doctors, too, report, that their patients have been having a hard experience lately. What with influenza on the one hand and a lean purse on the other, patients have found it very difficult to be patient. But business or no business, Christmas is Christmas, and the lean-pursed merchant, the hard-toiling doctor, the impatient patient, the out-o'-work, and the very waif of the street, all make up their minds to a simultaneous cremation of their past troubles, and to an united, happy, feasting, and merry-making time at the Christmas season.

Merry-making is fine medicine. Nobody knows how many incipient illnesses are cut short, how many break-downs of physiological energy are prevented by the brisk holiday, the good eating, the cheery fellowship, the "go" and "abandon" of Christmastide. What the long, dreary English winter would be without it one can hardly picture. But with it dull November is forgotten, December is victoriously conquered, and new energies are stored up wherewith to resist the onslaughts of January, February, and bleak March.

Some people complain that Christmas, which is a religious feast, is turned into an occasion of mere secular eating, drinking, and making merry. To our thinking, the main idea of Christmas is that of joyfulness; and a people so physically robust as ourselves, so little metaphysical, must express their joy in their own way. The Englishman is a large-hearted, tolerant, neighbour-loving creature, and to him a generous feast, and the free flow of happy conversation with his neighbours and old friends, is much more of a consecrating occasion than many a religious service to a more fanatical but less wholesome-hearted man. The founder of Christianity, whose birth we celebrate on Christmas Day, put forward children as the type of human creature which He best loved. Now children are never fanatical; they love play, and innocent joys, and cakes, and physical contentments. Their elders, at Christmas time, mostly conduct themselves like grown-up children let out from a very stern school; and in our judgment they are doing a very "Christian" thing when they so act. Christianity has absolutely nothing at all in common with sour fanaticism. It is, beyond all other faiths, the religion of earth and time, as well as of heaven and eternity.

Christmas cheer, after all, need not be made indigestible. When it is looked at a little in detail the analysis is decidedly reassuring. A fine plump turkey presents a noble figure, and two or three good slices from the breast are almost as easy of digestion as an oyster or a sweetbread. Even the goose, if he be well cooked, and eaten with apple sauce, can be disposed of without qualms by the fairly healthy stomach. As for the "Roast Beef of Old England," the honourable and honoured "Sir Loin," the miserablest anchorite and dyspeptic in the world may warm the poor fires of digestion with a slice from his incomparable upper cut. The very plum pudding of modern times, though not less "rich" than that of our grandmother's days, is so deftly compounded by the modern cook, and so thoroughly boiled, that dyspepsia itself may well be content to try a helping. Then the apples and the nuts, the raisins and the almonds, the oranges and the wine—what are these but light and cheerful aids to that beaming and happy frame of mind in which digestion proceeds as merrily as the song of the lark when he rises from his couch of primroses to greet the dawn of the spring morning.

Be pleased to remember, critics all, that the whole world was not made for dyspeptics, or even for the lovers of tracts and physic. It is entirely certain, notwithstanding our innumerable religious and medical books and newspapers, that there are large numbers of quite respectable and deserving people who never take physic and who never read tracts. Because some of you have refractory digestions, are these good people to have no Christmas "cakes and ale?" Besides, what, oh what would the children do if there were no Christmas holidays, no presents, no plum pudding, and no games? An answer to this question is not even possible. When one comes to think of it, Christmas is, above all things, "the Feast of the Children" in England. And how peculiarly appropriate this is we can see at a glance. The birth and childhood of the "Light of the World" we celebrate on Christmas Day. What can be so altogether fitting as that the whole Christmas period should be a period of beautiful home sunshine for the child world? Romp away, children; eat your plum pudding, and shout, and make a noise. You, at least, can do these things with innocent and joyous hearts, whatever may be the condition of the consciousness of your elders. There ought to be no dash of regret or melancholy in the full bumper of Christmas cheer; and that there might not be, we have all, no doubt, by this time, made some effort more or less considerable to send the warmth of the Christmas fire and a share of the Christmas feast into some home or lodging-place, which without us would have known no Christmas delights. We do not make any appeal for the distressed poor for the present Christmas, because we take it for granted that all our own readers are well-conditioned readers, and have done their duty like men and women and wholesome children long before this twenty-third of December. But in case any poor dependent has been forgotten, let us remind the forgetful that to the poor Christmas is one of the brightest spots in the year, when their betters remember their neighbourly love and duty; and, alas! it may be, and no doubt often is, one of the most cold, dreary, and disappointing of all seasons when that neighbourly love and duty are neglected by the prosperous. What we want to be assured of is that every man, woman, and child in our happy country, among the poorest poor as well as among the most prosperous rich, shall wake up on the dawn of the twenty-fifth of December singing "Christians, Awake, Salute the Happy Morn."

